# Characterization of the gila monster (*Heloderma suspectum suspectum*) venom proteome

**DOI:** 10.1016/j.dib.2015.01.007

**Published:** 2015-02-13

**Authors:** Kristian W. Sanggaard, Thomas F. Dyrlund, Line R. Thomsen, Tania A. Nielsen, Lars Brøndum, Tobias Wang, Ida B. Thøgersen, Jan J. Enghild

**Affiliations:** a Department of Molecular Biology and Genetics, Aarhus University, Denmark; b Interdisciplinary Nanoscience Center, Aarhus University, Denmark; c Museum of Natural History, Aarhus University, Denmark; d Department of Zoophysiology, Aarhus University, Denmark

## Abstract

The data presented here is related to the research article entitled “Characterization of the gila monster (*Heloderma suspectum suspectum*) venom proteome” by Sanggaard et al. in Journal of Proteomics [1]. The gila monster venom was collected, analyzed by 2D-gel electrophoresis and after Coomassie-Brilliant Blue staining the major spots were excised, subjected to in-gel trypsin digestion, and analyzed by LC–MS/MS. Subsequently, the venom proteins were identified based on *de novo* sequencing and homology searching. The mass spectrometry proteomics data have been deposited to the ProteomeXchange (dataset identifier PXD0001343), and in the present article we present an overview of the identified proteins. Protein identification failed for three of the selected spots, with the method described above. Instead, an iterative process, based on *de novo* sequencing, was employed.

**Specifications Table**

**Table:** 

Subject area	*Biology*
More specific subject area	*Venomics*
Type of data	*Figure and Tables*
How data was acquired	*Mass spectroscopy using an AB Sciex TripleTOF 5600^+^instrument*
Data format	*Processed*
Experimental factors	*2D –PAGE, in-gel trypsin treatment and micropurification of tryptic peptides*
Experimental features	*Tryptic peptides were analyzed by LC-MS/MS. PEAKS Studio 7.0 was employed for subsequent de novo sequencing and homology searching*
Data source location	*Jutland, Denmark (Danish breeder of gila monsters and beaded lizards)*
Data accessibility	*The data is accessible via this article, via the related research article*[1]*, and at the ProteomeXchange consortium* (*http://proteomecentral.proteomexchange.org*) *via the PRIDE partner repository with the dataset identifier PXD0001343*[2,3].

**Value of the data**

• The data present the first global protein characterization of lizard venom
• The data provides insights in the function and evolution of lizard venom
• The data facilitates identification of 19 novel helodermatid venom proteins
• The study presents a novel iterative *de novo* sequencing-based approach for identification of proteins from species with unknown genomes.

## 1 Experimental design

**Fig. 1:**
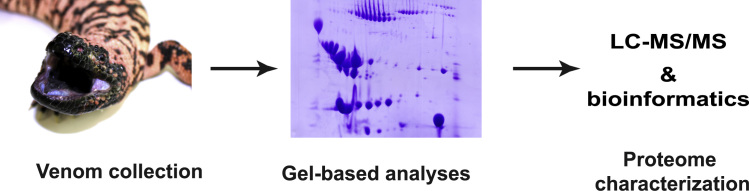
The workflow Diagram illustrates the study׳s experimental design.

The aim of this project was to provide a global overview of the protein composition in venom from helodermatids, the archetypical venomous lizard species including beaded lizards and gila monsters (Fig. 1). Initially venom samples were collected from four species and the overall protein composition was assed by SDS-PAGE. Subsequently a more detailed and targeted analysis of the venom proteome was performed focusing on the reticulated gila monster (*Heloderma suspectum suspectum*). Here the proteome was analyzed by 2D –PAGE followed by in-gel trypsin treatment of 58 selected spots. The resulting tryptic peptides were micropurified and subjected to LC–MS/MS analyses. Finally the obtained data were interrogated by PEAKS studio 7.0, which facilitates *de novo* sequencing and cross-species homology searching [4,5], in order to identify the gila monster venom proteome (Fig. 1). In addition, an iterative *de novo* sequencing-based method was developed for the analyses of LC–MS/MS data, which did not result in protein identification by the initial analyses by PEAKS.

## 2 Materials

Chemicals were from Sigma Aldrich, MS-grade trypsin was from Promega, and StageTips (C18) were from Thermo Scientifics. 2-D Clean-up kit, 2-D Quant Kit, 7 cm Immobiline DryStrip Gels, and IPG buffer were from GE Healthcare.

## 3 Methods and data

### 3.1 Venom collection

Venom milking was performed in collaboration with a Danish breeder. The animals were housed in a vivarium, fed rodents or chickens once every 7–14 days, and fresh water was always available. Venom was collected from male individuals of the following species: Reticulated gila monster (*Heloderma suspectum suspectum*), banded gila monster (*Heloderma suspectum cinctum*), Rio Fuerte beaded lizard (*Heloderma exasperatum*), and Mexican beaded lizard (*Heloderma horridum*). The lizards were enticed to bite repeatedly on a soft rubber tube and the venom glands were then gently massaged to facilitate the release of venom that subsequently was pipetted directly from the mouth. Venom samples contaminated with blood were discarded. After collection, a freshly prepared protease inhibitor cocktail was added and the samples were placed on dry ice, and later moved to an −80 °C freezer for storage.

### 3.2 Gel electrophoresis

**Fig. 2:**
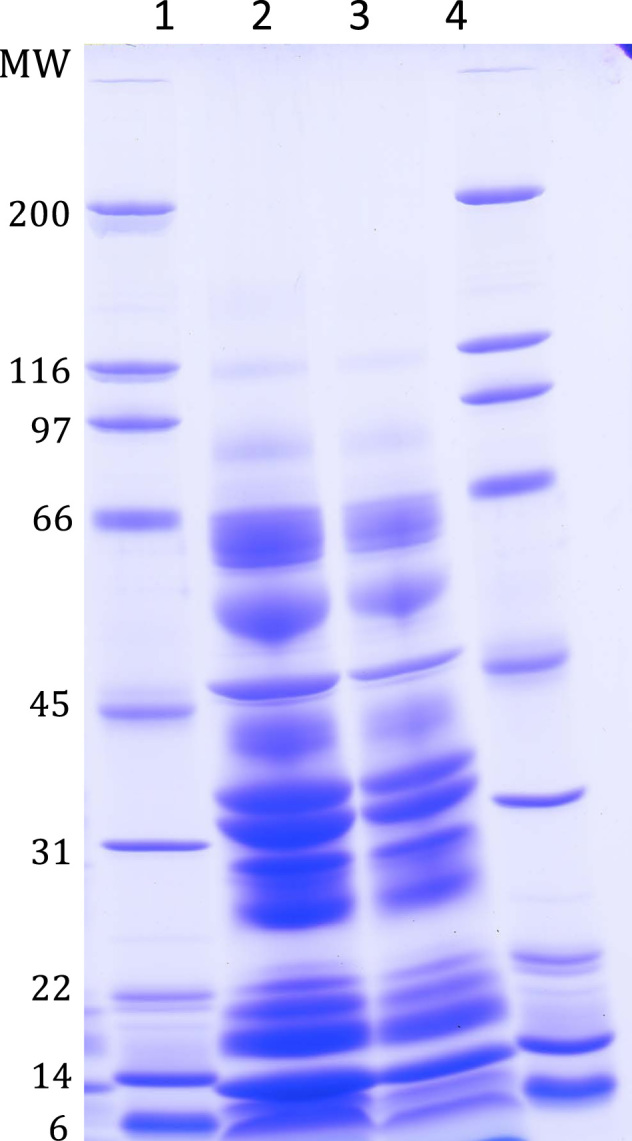
Coomassie Brilliant Blue-stained SDS-PAGE analysis Of venom From reticulated gila Monster (*Heloderma Suspectum suspectum*) (lane 2) And banded gila Monster (*Heloderma Suspectum cinctum*) (lane 3). The molecular weights of the proteins in the molecular weight standards (lane 1 And lane 4) are Shown in kDa. The analysis shows that the overall protein composition of the venom proteins in the two analyzed subspecies is similar. The collected venom samples were analyzed without prior protein precipitation. This is in contrast to the data presented in the accompanying Journal of Proteomics article by Sanggaard et al. However, the venom proteins display similar migration patterns in the two analyses. It indicates That major gila monster venom proteins are not lost by introducing a protein precipitation step prior To SDS-PAGE.

Initially, the venom from the four species was analyzed by sodium dodecyl sulphate-polyacrylamide gel-electrophoresis (SDS-PAGE). In details, approximately 30 μl of venom was centrifuged to remove particular matters, the proteins in the supernatant were recovered by ethanol precipitation, the resulting pellet was dissolved in 60 μl TBS, approximately 1 μl was transferred to SDS-sample-buffer containing dithiothreitol (DTT), the samples boiled, subjected to SDS-PAGE using 5–15% gradient gels [6], and finally the resolved proteins were stained using Coomassie Brilliant Blue (Fig. 1 in the Journal of Proteomics paper [1]). In order to evaluate whether the precipitation step significantly changes the protein composition, the venom from gila monsters was also analyzed directly by SDS-PAGE, without ethanol precipitation (Fig. 2). Comparison of the two SDS-PAGE analyses suggests that the precipitation step does not affect the overall protein composition.

To facilitate a deeper analysis of the composition of helodermatid venom, the collected venom from the reticulated gila monster was subjected to 2D –PAGE. The detailed protocol for the 2D –PAGE analyses is described in the paper published in Journal of Proteomics [1]. In short, venom from one individual was analyzed on 2D-gels with isoelectric focusing from either pH 3–10 or from pH 4–7, and after the electrophoresis the resolved proteins were stained using Coomassie Brilliant blue (see Fig. 2 in the Journal of Proteomics paper [1]).

### 3.3 In-gel trypsin treatment and LC-MS/MS analyses

The major spots, from the 2D-gel with an isoelectric focusing from pH 4–7, were excised and subjected to in-gel trypsin treatment, essential as described [7]. Prior to the LC-MS/MS analyses the resulting tryptic peptides were micro-purified using C_18_ stageTips. Subsequently, the samples were injected, trapped and desalted isocratically on a pre-column (Biosphere C18, ID 100 µm×2 cm, 5 µm, 120 Å, NanoSeparation, Nieuwkoop, Netherlands) whereupon the peptides were eluted onto and separated by a 15 cm analytical column (75 μm i.d.), pulled in-house (P2000 laser puller, Sutter Instrument Co.), and packed with ReproSil-Pur C18-AQ 3 μm resin (Dr. Marisch GmbH, Ammerbuch-Entringen, Germany). Peptides were eluted at a flow rate of 250 nl/min using a 50 min gradient from 5% to 35% B (0.1% formic acid, 90% acetonitrile). The samples were run multiple times at different concentrations to assure that the optimal amount of the samples were loaded. The liquid chromatography was performed on a nano flow HPLC system (Thermo Scientific, EASY-nLC II) connected directly to the mass spectrometer (AB Sciex TripleTOF 5600^+^), which was equipped with a NanoSpray III source (AB Sciex) and operated under Analyst TF 1.6.0 control. The LC-MS/MS runs that did not initially result in protein identification, were re-analyzed, the data merged, and the merged file used for the PEAKS-depended protein identification, as described below. The mass spectrometry proteomics data have been deposited to the ProteomeXchange Consortium (http://proteomecentral.proteomexchange.org) via the PRIDE partner repository (dataset identifier PXD0001343) [2,3].

### 3.4 Protein identification

*Sauropsida* (birds, crocodiles, lizards, snakes, and turtles) protein sequences were extracted from the Swiss-Prot database version 2014_06 (5.913 sequences) and 890.360 *Sauropsida* protein sequences were extracted from NCBI on June 11, 2014. The two sets of sequences were merged into a single *Sauropsida* protein database containing a total of 896.273 sequences. The collected MS files were converted to Mascot generic format (MGF) using the AB SCIEX MS Data Converter beta 1.3 (AB SCIEX) and the “ProteinPilot MGF” parameters. Finally, the peak lists were used to interrogate the in-house constructed *Sauropsida* protein database. For these analyses PEAKS Studio 7.0 was employed using trypsin as the enzyme with one missed cleavage allowed. Carbamidomethyl was entered as a fixed modification and PEAKS׳ PTM algorithm was applied to identify variable modifications [8]. The mass accuracy of the precursor and product ions was set to 10 ppm and 0.2 Da, respectively, the instrument was specified as an AB Sciex TripleTOF and fragmentation method as collision-induced dissociation. PEAKS was used to perform *de novo* sequencing [9], database searches [9], PTM analyses [8], and the finally homology searches were performed using the SPIDER algorithm [4]. The SPIDER algorithm allows the matching of *de novo*-obtained amino acid sequences with homologous protein sequences in the database [5]. After the PEAKS analyses the resulting list of proteins were filtered using a peptide false discovery rate of 0.1% and only proteins identified with a minimum of 3 peptides are reported. All protein identifications, inclusive PTMs, supporting peptides, and spectral counts, are available online in the PRIDE archive (PXD001343). In order to simplify the data (Supplementary Table 1), keratins were subsequently removed from the protein lists, and protein groups (based on shared identified peptides) were combined into one hit based on the following criteria: (1) The highest scoring protein hit was kept, except that Swiss-Prot accessions were prioritized over NCBI accessions, and (2) Proteins with three or more unique peptides were not grouped.

### 3.5 Iterative d*e novo-*sequencing-based approach for protein identification

The procedure described above failed to identify the proteins in spots 19, 21, and 54, possibly due to a low degree of sequence similarity between the gila monster protein sequences and the available deposited protein sequences. However, by changing the parameters in PEAKS to allow *de novo* peptide sequence tags, spot 54 was identified as phospholipase A_2_ (type III), but with a low score, inconsistent with the staining intensity of the 2D-gel spot. For spot 19 and 21 the *de novo* amino acid sequences with average local confidence scores above 90% were extracted from PEAKS, and searched against the sequence database nrdb95 using MS-BLAST and standard settings. This software is similar to the SPIDER function in PEAKS and was developed to identify homologous proteins using sequence obtained by *de novo* amino acid sequencing [10]. The MS-BLAST analyses of the two sets of amino acid sequences of peptides identified kallikrein-like toxin sequences originating from other lizard species. The amino acid sequences of 33 proteins were then extracted from the NCBInr database using the combined search criteria “toxin and lizard and kallikrein”. Subsequently, the PEAKS׳ SPIDER function was used to interrogate these 33 extracted protein sequences with the LC-MS/MS data from the two relevant samples. The highest scoring kallikrein-like toxin homologous from each of these searches indicated the most likely content of the two 2D gel spots although the protein identification scores were, similar to the identification of phospholipase A_2_ (type III) in spot 54, low. In order to substantiate the findings we extracted the most confident amino acid sequences of peptides obtained by *de novo* sequencing by the PEAKS׳ analyses of the three LC-MS/MS dataset, and compared these sequences with the weakly identified homologous proteins, as described in details below.

The PEAKS-generated amino acid sequences with average local confidence scores above 90% were extracted and only individual amino acid residues with a total local confidence score of minimum 90% was accepted. The extracted *de novo*-obtained amino acid sequences were subsequently manually filtered: Peptides, which were not identified with a minimum of 4 spectral counts, and peptides, which did not include a sequence of minimum of 5 consecutive residues, were removed. Afterwards, the remaining amino acid sequences in each of the three analyses were merged if they had an overlapping stretch of at least 4 identical amino acid residues. If disagreements existed in the sequences to be combined, the consensus sequence was derived from the sequence with the highest number of spectral counts. Using the described approach the final list of *de novo*-derived peptides sequences from the three LC-MS/MS analyses included 10, 11, and 30 peptides for respectively spot 19, 21, and 54 (Supplementary Table 2). The lists of peptides were then aligned with the template sequences (the two kallikrein-like proteases and the type III phospholipase A_2_, respectively), and if the peptides aligned (defined by minimum 4 identical residues) with the homologous protein, the sequence of the homologous protein was changed according to the *de novo-*obtained peptide sequence. For spot 19, 4 out of the 10 *de novo*-obtained amino acid sequences aligned with the kallikrein-like protease sequence, for spot 21 the numbers were 5 out of 11, and for spot 54 all 30 peptides aligned with the type III phospholipase A_2_ (Supplementary Table 2). This relative high similarity (especially for spot 54) between the unbiased extracted *de novo*-obtained amino acid sequences and the weakly identified proteins, suggest that these proteins are indeed present in the three analyzed 2D gel spots. Subsequently, PEAKS׳ SPIDER function was applied to search the LC-MS/MS data against the new optimized sequences with the same search and filtering criteria as described in Section 3.4. Confident mutations, suggested by SPIDER, were subsequently included in the modified sequences. Finally, the resulting three protein sequences were included in the *Sauropsida* sequence database, and the three samples were reanalyzed using PEAKS. The resulting protein identifications are included in Supplementary Table 1.

## Footnotes

Appendix A Supplementary data associated with this article can be found in the online version at doi:10.1016/j.dib.2015.01.007.
