# Supplementary material for: Characterization of the gila monster (Heloderma suspectum suspectum) venom proteome
Source: Data Brief. 2015 Feb 13;3:137–42. doi: 10.1016/j.dib.2015.01.007 (PMC4510068; doi:10.1016/j.dib.2015.01.007)
Supplement: Supplementary file 1 — Supplementary data [file mmc1.pdf]

Table 1:

Spot #	Accession Nr	Organism	Protein Name	Mass kDa	Score -10lgP
1	gi|190700985	Heloderma suspectum cinctum	Hyaluronidase toxin	16,195	178.91
2	gi|190700985	Heloderma suspectum cinctum	Hyaluronidase toxin	16,195	173.68
	gi|558142188	Pelodiscus sinensis	PREDICTED: neuroendocrine convertase 1 isoform X3	69,928	104.45
3	gi|637260392	Anolis carolinensis	PREDICTED: neuroendocrine convertase 1	83,573	236.67
	gi|190700985	Heloderma suspectum cinctum	Hyaluronidase toxin	16,195	147.21
	P80003	Heloderma suspectum	Acidic phospholipase A2 PA4	15,640	106.89
	C6EVG7	Heloderma suspectum cinctum	Natriuretic and helokinestatin peptides	19,672	88.40
4	gi|637304799	Anolis carolinensis	PREDICTED: semaphorin-3E isoform X2	89,508	241.25
5	gi|637304799	Anolis carolinensis	PREDICTED: semaphorin-3E isoform X2	89,508	176.53
6	gi|637381731	Anolis carolinensis	PREDICTED: LOW QUALITY PROTEIN: myeloperoxidase-like	80,855	139.54
7	gi|637270817	Anolis carolinensis	PREDICTED: hepatocyte growth factor-like protein	80,320	227.65
8	gi|190700987	Heloderma suspectum cinctum	Kallikrein toxin 1	28,229	144.63
	gi|449280893	Columba livia	Ovochymase-2, partial	26,050	86.33
9	gi|637304803	Anolis carolinensis	PREDICTED: semaphorin-3E isoform X2	82,875	159.24
10	gi|47604946	Gallus gallus	Thymosin, beta 4	5,181	123.32
	gi|449278384	Columba livia	Glyceraldehyde-3-phosphate dehydrogenase, partial	34,982	114.24
11	gi|432585	Heloderma horridum	Gilatoxin=kallikrein-like serine protease	26,845	139.18
12	gi|432585	Heloderma horridum	Gilatoxin=kallikrein-like serine protease	26,845	109.89
13	gi|432585	Heloderma horridum	Gilatoxin=kallikrein-like serine protease	26,845	331.01
	P80003	Heloderma suspectum	Acidic phospholipase A2 PA4	15,640	126.93
	gi|558133676	Pelodiscus sinensis	PREDICTED: kallikrein-11-like	25,956	118.77
	gi|300872907	Celestus warreni	Kallikrein-Cwar3	26,815	83.33
14	gi|432585	Heloderma horridum	Gilatoxin=kallikrein-like serine protease	26,845	280.78
	gi|300872907	Celestus warreni	Kallikrein-Cwar3	26,815	117.63
	gi|558133676	Pelodiscus sinensis	PREDICTED: kallikrein-11-like	25,956	112.37
15	gi|432585	Heloderma horridum	Gilatoxin=kallikrein-like serine protease	26,845	343.38
	gi|300872907	Celestus warreni	Kallikrein-Cwar3	26,815	125.50
	gi|558133676	Pelodiscus sinensis	PREDICTED: kallikrein-11-like	25,956	122.81
16	gi|432585	Heloderma horridum	Gilatoxin=kallikrein-like serine protease	26,845	205.52
	gi|300872907	Celestus warreni	Kallikrein-Cwar3	26,815	143.45
	gi|387014304	Crotalus adamanteus	14-3-3 protein sigma	27,929	126.41
	C6EVG7	Heloderma suspectum cinctum	Natriuretic and helokinestatin peptides	19,672	123.53
	P80003	Heloderma suspectum	Acidic phospholipase A2 PA4	15,640	121.53
17	gi|327268290	Anolis carolinensis	PREDICTED: peroxiredoxin-4	30,492	99.84
18	gi|591387951	Chelonia mydas	PREDICTED: phosphatidylethanolamine-binding protein 4	23,679	121.06
	gi|537459604	Micrurus fulvius	Phosphatidylethanolamine-binding protein 4	23,247	92.50
19	gi|300872895-Mod	Gerrhonotus infernalis	Kallikrein-Ginf2	27,247	283.57
20	C6EVG7	Heloderma suspectum cinctum	Natriuretic and helokinestatin peptides	19,672	101.62
21	gi|300872907-Mod	Celestus warreni	Kallikrein-Cwar3	26,923	217.88
	gi|300872895-Mod	Gerrhonotus infernalis	Kallikrein-Ginf2	27,247	134.81
22	gi|190700979	Heloderma suspectum cinctum	CRISP toxin	27,512	264.38
23	D2X5W4	Heloderma suspectum cinctum	Helofensin-3	19,456	253.69
24	P80003	Heloderma suspectum	Acidic phospholipase A2 PA4	16,696	143.14
25	gi|432585	Heloderma horridum	Gilatoxin=kallikrein-like serine protease	26,845	120.10
	P80003	Heloderma suspectum	Acidic phospholipase A2 PA4	16,696	113.26
26	gi|432585	Heloderma horridum	Gilatoxin=kallikrein-like serine protease	26,845	83.12
27	gi|190700997	Heloderma suspectum cinctum	Type III phospholipase A2 toxin 1	16,696	181.56
	gi|530578376	Chrysemys picta bellii	PREDICTED: rho GDP-dissociation inhibitor 1	23,234	114.92
	gi|602668510	Python bivittatus	PREDICTED: semaphorin-3E-like, partial	72,042	104.52
28	gi|151176139	Anas platyrhynchos	Beta-actin	41,738	130.86
	P80003	Heloderma suspectum	Acidic phospholipase A2 PA4	16,696	85.54
29	gi|602668510	Python bivittatus	PREDICTED: semaphorin-3E-like, partial	72,042	166.44
	P80003	Heloderma suspectum	Acidic phospholipase A2 PA4	16,696	164.96
	gi|432585	Heloderma horridum	Gilatoxin=kallikrein-like serine protease	26,845	99.70
30	gi|190700997	Heloderma suspectum cinctum	Type III phospholipase A2 toxin 1	16,696	227.19
	gi|637304803	Anolis carolinensis	PREDICTED: semaphorin-3E isoform X2	82,875	182.88
	gi|432585	Heloderma horridum	Gilatoxin=kallikrein-like serine protease	26,845	89.37
31	gi|190700997	Heloderma suspectum cinctum	Type III phospholipase A2 toxin 1	16,696	192.26
	gi|637304803	Anolis carolinensis	PREDICTED: semaphorin-3E isoform X2	82,875	122.46
	gi|432585	Heloderma horridum	Gilatoxin=kallikrein-like serine protease	26,845	75.80

32	gi|190700997	Heloderma suspectum cinctum	Type III phospholipase A2 toxin 1	16,696	216.92
	gi|637304803	Anolis carolinensis	PREDICTED: semaphorin-3E isoform X2	82,875	126.00
	C6EVG7	Heloderma suspectum cinctum	Natriuretic and helokinestatin peptides	19,672	99.17
	gi|432585	Heloderma horridum	Gilatoxin=kallikrein-like serine protease	26,845	71.88
33	gi|530582705	Chrysemys picta bellii	PREDICTED: phosphatidylethanolamine-binding protein 4	23,679	115.95
34	D2X5W4	Heloderma suspectum cinctum	Helofensin-3	19,456	302.55
	P80003	Heloderma suspectum	Acidic phospholipase A2 PA4	16,696	81.42
35	P16354	Heloderma suspectum	Phospholipase A2 isozymes PA3A/PA3B/PA5	16,048	426.56
	gi|190700999	Heloderma suspectum cinctum	Type III phospholipase A2 toxin 2	18,034	365.53
	C6EVG7	Heloderma suspectum cinctum	Natriuretic and helokinestatin peptides	19,672	105.43
36	P16354	Heloderma suspectum	Phospholipase A2 isozymes PA3A/PA3B/PA5	16,048	417.89
	C6EVG7	Heloderma suspectum cinctum	Natriuretic and helokinestatin peptides	19,672	139.44
37	gi|190700997	Heloderma suspectum cinctum	Type III phospholipase A2 toxin 1	16,696	385.56
	C6EVG7	Heloderma suspectum cinctum	Natriuretic and helokinestatin peptides	19,672	163.49
38	gi|190700997	Heloderma suspectum cinctum	Type III phospholipase A2 toxin 1	16,696	298.36
	C6EVG7	Heloderma suspectum cinctum	Natriuretic and helokinestatin peptides	19,672	92.40
39	P80003	Heloderma suspectum	Acidic phospholipase A2 PA4	16,696	332.93
	C6EVG7	Heloderma suspectum cinctum	Natriuretic and helokinestatin peptides	19,672	86.98
40	P80003	Heloderma suspectum	Acidic phospholipase A2 PA4	16,696	286.28
	C6EVG7	Heloderma suspectum cinctum	Natriuretic and helokinestatin peptides	19,672	144.55
41	P80003	Heloderma suspectum	Acidic phospholipase A2 PA4	16,696	370.82
42	P80003	Heloderma suspectum	Acidic phospholipase A2 PA4	16,696	183.45
	C6EVG7	Heloderma suspectum cinctum	Natriuretic and helokinestatin peptides	19,672	127.03
43	P80003	Heloderma suspectum	Acidic phospholipase A2 PA4	16,696	232.86
	gi|432585	Heloderma horridum	Gilatoxin=kallikrein-like serine protease	26,845	86.06
44	P80003	Heloderma suspectum	Acidic phospholipase A2 PA4	16,696	197.99
	C6EVG7	Heloderma suspectum cinctum	Natriuretic and helokinestatin peptides	19,672	124.58
45	P80003	Heloderma suspectum	Acidic phospholipase A2 PA4	16,696	87.93
46	gi|190700997	Heloderma suspectum cinctum	Type III phospholipase A2 toxin 1	16,696	164.37
	gi|602627628	Python bivittatus	PREDICTED: myelomonocytic growth factor-like	27,726	77.90
47	gi|190700997	Heloderma suspectum cinctum	Type III phospholipase A2 toxin 1	16,696	157.93
	Q3HXY8	Pseudonaja textilis	Venom nerve growth factor 2	27,472	108.62
48	gi|408385878	Abronia graminea	VEGF-Abr-1	22,330	175.37
	P16354	Heloderma suspectum	Phospholipase A2 isozymes PA3A/PA3B/PA5	16,048	139.73
49	P80003	Heloderma suspectum	Acidic phospholipase A2 PA4	16,696	101.92
	C6EVG7	Heloderma suspectum cinctum	Natriuretic and helokinestatin peptides	19,672	97.42
	gi|408385878	Abronia graminea	VEGF-Abr-1	22,330	71.86
50	gi|190700997	Heloderma suspectum cinctum	Type III phospholipase A2 toxin 1	16,696	171.09
51	P80003	Heloderma suspectum	Acidic phospholipase A2 PA4	16,696	151.51
52	P80003	Heloderma suspectum	Acidic phospholipase A2 PA4	16,696	101.01
53	P80003	Heloderma suspectum	Acidic phospholipase A2 PA4	16,696	186.30
	P04204	Heloderma suspectum	Exendin-2-long	9,531	60.83
54	gi|408385876-Mod	Abronia graminea	PLA2-Abr-1	36,784	460.12
	P04204	Heloderma suspectum	Exendin-2-long	9,531	165.16
55	P04204	Heloderma suspectum	Exendin-2-long	9,531	160.82
56	C6EVG1	Heloderma suspectum cinctum	Exendin-4	9,479	190.00
	P16354	Heloderma suspectum	Phospholipase A2 isozymes PA3A/PA3B/PA5	16,048	183.31
	P20394	Heloderma horridum	Exendin-3	9,481	147.31
57	P04204	Heloderma suspectum	Exendin-2-long	9,531	165.16
	P80003	Heloderma suspectum	Acidic phospholipase A2 PA4	16,696	90.54
58	gi|248418	Heloderma suspectum	Exendin-4	4,188	196.42
	P20394	Heloderma horridum	Exendin-3	9,481	143.91
	C6EVG7	Heloderma suspectum cinctum	Natriuretic and helokinestatin peptides	19,672	138.04

Table 1. The protein composition of the 58 analyzed 2D-gel spots. The table shows the proteins identified in each of the 58 spots using PEAKS to interrogate a database composed of protein sequences from birds, crocodiles, lizards, snakes and turtles (*sauropsida)* with the obtained LC-MS/MS data. Keratins have been removed from the list. In order to simplify the data, protein groups (based on shared identified peptides) were combined into one hit based on the following criteria: 1) The highest scoring protein hit was kept, except that Swiss-Prot accessions were prioritized over NCBI accessions, and 2) Proteins with three or more unique peptides were not grouped. The full list of identified proteins (without grouping and removal of keratins) has been deposited to the ProteomeXchange Consortium via the PRIDE partner repository with the dataset identifier PXD001343.
