# Supplementary material for: Characterization of the gila monster (Heloderma suspectum suspectum) venom proteome
Source: Data Brief. 2015 Feb 13;3:137–42. doi: 10.1016/j.dib.2015.01.007 (PMC4510068; doi:10.1016/j.dib.2015.01.007)
Supplement: Supplementary file 2 — Supplementary data [file mmc2.pdf]

Table 2:

Identification of three gila monster venom proteins using de novo sequencing and partial reconstruction of sequences. The tables and alignments below describe how the protein content of spot 19, 21, and 54 have been identified using an iterative process based on de novo sequencing performed by PEAKS (please see method section for further details). Alignments of the original sequences with the user-modified sequences are included. **The tables include the following footnotes:**

1: Peptide sequences with Average Local Confidence (ALC) scores above 90% and Total Local Confidence (TLC) scores above 90%. If the ALC score for a peptide sequence is above 90%, but the TLC score at certain positions is below 90%, the peptide sequence is included in the table and the mass of the unassigned part of the sequence (TLC < 90%) is displayed inside bracket parentheses. The masses displayed in the ordinary parentheses indicate a modification. Sequences shorter than five amino acid residues are not included.

2: The parentheses have been removed and sequence-tags N- or C-terminal to the bracket parentheses have been removed unless the tags are five amino acid residues or more.

3: Peptide sequences with less than 4 spectral counts have been removed.

4: Modified protein sequences were constructed based on the template protein sequences and the obtained de novo peptide sequences, as described in the method section. Here, an alignment of the resulting modified sequences with the original template sequences are shown. The red part of the sequences highlights the part of the modified sequences, which, compared to the template, were changed according to the de novo peptide sequences. Confident mutations suggested by the SPIDER algorithm in PEAKS are displayed in green.

Spot 19

Sequences obtained from PEAKS[1]	Cleaned sequences[2]	Spectral counts[3]
GPGLYTR	GPGLYTR *	27
C(+57.02)SVM(+15.99)GWGSLTTSK	CSVMGWGSLTTSK *	16
LSSPATL[201.1]R	LSSPATL	11
VSAGTM(+15.99)C(+57.02)Y[97.1]DTASTTK	VSAGTMCY DTASTTK *	10
VVTVSL[97.1]R	VVTVSL	9
DSC(+57.02)PDFTADLM(+15.99)M(+15.99)LK	DSCPDFTADLMMLK *	8
LATVL[87.0]PR	LATVL	5
LSSPATL[114.0]SR	LSSPATL	4
LATVL[184.1]R	LATVL	4
VATVSL[97.1]R	VATVSL	4

* Peptide sequences that align with the homology-based protein hit. These sequences were used to construct the modified protein sequence.

Alignment[4]

```
1     MEPAKLLALLLLLLPSLVSAHHKRILGGKECNEAEHPWLVLLYESKKPFCSGILLDRNWV    60   gi|300872895|gb|ADK39260.1|
1     MEPAKLLALLLLLLPSLVSAHHKRILGGKECNEAEHPWLVLLYEPKKPFCSGILLDRNWV    60   gi|300872895-Modified
      ******************************************** ***************

61    VTAAHCYVSGEIEIRLGLHNRNMPRGDEQMRVSAATLCYPDTASTTQNSCADFTADIMMI   120   gi|300872895|gb|ADK39260.1|
61    VTAAHCYVSGEIEIRLGLHNRNMPRGDEQMRVSAGTMCYPDTASTTKDSCPDFTADLMML   120   gi|300872895-Modified
      **********************************.*:*********::** *****:**:

121   KLNSPVKYSKHIGPLLLPTGSVFVGTKCRVMGWGTTTTSEVTFPIVPQCANINILENRVC   180   gi|300872895|gb|ADK39260.1|
121   KLNSPVKFNKHIGPLLLPTGSVFVGTKCSVMGWGSLTTSKVTFPIVPQCANINILENRVC   180   gi|300872895-Modified
      *******:.******************* *****: ***:********************
```

```
181   AVAYPSWRMTNDMLCAGDQKGCKDTCQGDSGGPLICGGQLQGIISMGKSPGPGVYTNVNS  240  gi|300872895|gb|ADK39260.1|
181   AVAYPSWRMTNDMLCAGDQKGCKDTCQGDSGGPLICGGQLQGIISMGKSPGPGLYTRVNS  240  gi|300872895-Modified
      *****************************************************:**.***

241   YFNWILEFI  249  gi|300872895|gb|ADK39260.1|
241   YFNWILEFI  249  gi|300872895-Modified
      *********
```

Spot 21

Sequences obtained from PEAKS[1]	Cleaned sequences[2]	Spectral counts[3]
LSAGTM(+15.99)C(+57.02)YPDTAR	LSAGTMCYPDTAR *	13
LLC(+57.02)AQLK	LLCAQLK	7
LSSPATLDSR	LSSPATLDSR	6
VTLPNVPHC(+57.02)[287.1]R	VTLPNVPHC *	6
VVTVSL[97.1]R	VVTVSL	5
DWVLTDK	DWVLTDK *	4
LSSPATLNSR	LSSPATLNSR	4
LC(+57.02)EM(+15.99)AYK	LCEMAYK	4
LSAGTM(+15.99)C(+57.02)YPDE[71.0]R	LSAGTMCYPDE *	4
LSSPATL[201.1]R	LSSPATL	4
LDAGTM(+15.99)C(+57.02)YPD[172.1]R	LDAGTMCYPD *	4

* Peptide sequences that align with the homology-based protein hit. These sequences were used to construct the modified protein sequence.

Alignment[4]

```
1     MEPIKLLAFLPLLLAFVSAKHKRIVGGQECSEDEHPWLVLFHDSTGPFCSGVLLDHNWVL  60  gi|300872907|gb|ADK39266.1|
1     MEPIKLLAFLPLLLAFVSAKHKRIVGGQECSEDEHPWLVLFHDSTGPFCSGVLLDHDWVL  60  gi|300872907-Modified
      ********************************************************:***

61    TAAHCYERGKMQMKLGVHNRNVLRGDEESRVSAAARCFPDRPGATQNSCEAFTADIMMVK  120  gi|300872907|gb|ADK39266.1|
61    TDKHCYERGKMQMKLGVHNRNVLRGDEESRLSAGTMCYPDTARATQNSCEAFTADIMMVK  120  gi|300872907-Modified
      *  ***************************:**.: *:**   *****************

121   LNSPVKYNKHISSLLLPSASVFVGAKCRVMGWGSNTVVRNTIVPHCVDLTILENRVCTAA  180  gi|300872907|gb|ADK39266.1|
121   LNLPVKYNKHISSLLLPSASVFVGAKCRVMGWGSNTVVTLPNVPHCVDLTILENRVCTAA  180  gi|300872907-Modified
      ** ***********************************    ******************

181   LPVLDMSDDDLCAGDPKGCKDSCQGDSGGPLVCDGQLQGIVSRGRSPGPGIYTKVDSYLN  240  gi|300872907|gb|ADK39266.1|
181   LPVLDMSDDDLCAGDPKGCKDSCQGDSGGPLVCDGQLQGIVSRGRSPGPGIYTKVDSYLN  240  gi|300872907-Modified
      ************************************************************

241   WILEFI  246  gi|300872907|gb|ADK39266.1|
241   WILEFI  246  gi|300872907-Modified
      ******
```

Spot 54

Sequences obtained from PEAKS[1]	Cleaned sequences[2]	Spectral counts[3]
LLAC(+57.02)ALR	LLACALR *	146
FLATLEA[128.1]K	FLATLEA *	95
[128.1]ALVVSAAEK	ALVVSAAEK *	84
AATC(+57.02)D[128.1]R	AATCD *	49
FLATLEAQK	FLATLEAQK *	44
AATC(+57.02)DQR	AATCDQR *	30
[154.1]GLAPVLVQSVWDANK	GLAPVLVQSVWDANK *	29
QALVVSAAEK	QALVVSAAEK *	22
[128.1]ALVVDAAEK	ALVVDAAEK *	18
VSAAEK	VSAAEK *	17
FLAELEA[128.1]K	FLAELEA *	15
[128.1]ALVVSA[71.0]E[128.1]	ALVVSA *	12
DQPAHTQWYAAT[275.1]QR	DQPAHTQWYAAT *	11

FLATLE[199.1]K	FLATLE *	9
[154.1]GLAPVLVQSVWDAG[57.0]K	GLAPVLVQSVWDAG *	9
LVVSAAEK	LVVSAAEK *	8
YAATC(+57.02)D[128.1]R	YAATCD *	8
[128.1]ALVVSA[71.0]EK	ALVVSA *	7
[340.1]AHTQADYAATC(+57.02)DQR	AHTQADYAATC *	6
DQPAHTQADYAAT[275.1]QR	DQPAHTQADYAAT *	6
[154.1]GLAPVLVQSVWDAG[185.1]	GLAPVLVQSVWDAG *	6
LEAQK	LEAQK *	6
[128.1]ALVVDA[71.0]EK	ALVVDA *	5
[128.1]ALVVSA[328.2]	ALVVSA *	5
ATLEAQK	ATLEAQK *	5
FLATLEA[256.2]	FLATLEA *	5
LAC(+57.02)ALR	LACALR *	5
DQPAHTQWYAATC(+57.02)DQR	DQPAHTQWYAATCDQR *	4
[154.1]GLAPVLVQDVWDANK	GLAPVLVQDVWDANK *	4
LATVL[184.1]R	LATVL *	4

* Peptide sequences that align with the homology-based protein hit. These sequences were used to construct the modified protein sequence.

Alignment[4]

```
1     MLASMASVGALLLAMNALVMPLASGSWSKDTTSSHTIAKGGRGRDRGQVLSFLWRRPRGL   60  gi|408385876|gb|AFU63217.1|
1     MLASMASVGALLLAMNALVMPLASGSWSKDTTSSHTIAKGGRGRDRGQVLSFLWRRPRGL   60  gi|408385876-Modified
      ************************************************************

61    PPVLVQSIWDPQGHLLACALRDEPAHTNWYLEAYKQQQALNFSGTELQHYLDTLEVQNDT  120  gi|408385876|gb|AFU63217.1|
61    APVLVQSVWDAN-KLLACALRDQPAHTQWYAATCDQRQALVVSAAEKQHFLATLEAQKDT  119  gi|408385876-Modified
       ******:**  :  :********:****:**    :  .*:***  .*..:*  **:*  ***.*:**

121   CWGAEYTGEDLDLGVRRTLALEEQEAQETNRIQRGLTMPGTLWCGAGNIASNFSHLGTFK  180  gi|408385876|gb|AFU63217.1|
120   CWGAEYTGEDLDLGVRRTLALEEQEAQETNRIQRGLTMPGTLWCGAGNIASNFSHLGTFK  179  gi|408385876-Modified
      ************************************************************

181   GPDMCCRDHDHCDIQISGLKYNYGVFNFRPHTISHCDCDTRFRNCLMSLSDSIADFIGKT  240  gi|408385876|gb|AFU63217.1|
180   GPDMCCRDHDHCDIQISGLKYNYGVFNFRPHTISHCDCDTRFRNCLMSLSDSIADFIGKT  239  gi|408385876-Modified
      ************************************************************

241   YFNVMFVPCFELKEGNACVEWNWMYMCTNYSQMPVAHLVDPSPYVPIGLPTPTLQPGTQS  300  gi|408385876|gb|AFU63217.1|
240   YFNVMFVPCFELKEGNACVEWNWMYMCTNYSQMPVAHLVDPSPYVPIGLPTPTLQPGTQS  299  gi|408385876-Modified
      ************************************************************

301   SQDRKRSQKKWKQLRHAHPNGAGQRRRLGR  330  gi|408385876|gb|AFU63217.1|
300   SQDRKRSQKKWKQLRHAHPNGAGQRRRLGR  329  gi|408385876-Modified
      ******************************
```
